# Stabilization of ε-iron carbide as high-temperature catalyst under realistic Fischer–Tropsch synthesis conditions

**DOI:** 10.1038/s41467-020-20068-5

**Published:** 2020-12-04

**Authors:** Shuai Lyu, Li Wang, Zhe Li, Shukun Yin, Jie Chen, Yuhua Zhang, Jinlin Li, Ye Wang

**Affiliations:** 1 Key Laboratory of Catalysis and Energy Materials Chemistry of Ministry of Education & Hubei Key Laboratory of Catalysis and Materials Science, South-Central University for Nationalities, Wuhan, 430074 China; 2 State Key Laboratory of Physical Chemistry of Solid Surfaces, Collaborative Innovation Center of Chemistry for Energy Materials, National Engineering Laboratory for Green Chemical Productions of Alcohols, Ethers and Esters, College of Chemistry and Chemical Engineering, Xiamen University, Xiamen, 361005 China

**Keywords:** Catalyst synthesis, Heterogeneous catalysis, Chemical engineering

## Abstract

The development of efficient catalysts for Fischer–Tropsch (FT) synthesis, a core reaction in the utilization of non-petroleum carbon resources to supply energy and chemicals, has attracted much recent attention. ε-Iron carbide (ε-Fe_2_C) was proposed as the most active iron phase for FT synthesis, but this phase is generally unstable under realistic FT reaction conditions (> 523 K). Here, we succeed in stabilizing pure-phase ε-Fe_2_C nanocrystals by confining them into graphene layers and obtain an iron-time yield of 1258 μmol_CO_ g_Fe_^−1^s^−1^ under realistic FT synthesis conditions, one order of magnitude higher than that of the conventional carbon-supported Fe catalyst. The ε-Fe_2_C@graphene catalyst is stable at least for 400 h under high-temperature conditions. Density functional theory (DFT) calculations reveal the feasible formation of ε-Fe_2_C by carburization of α-Fe precursor through interfacial interactions of ε-Fe_2_C@graphene. This work provides a promising strategy to design highly active and stable Fe-based FT catalysts.

## Abstract (web summary)

ε-Fe_2_C has been identified as the highly active phase for Fischer-Tropsch synthesis (FTS), but is stable only at low-temperature. Here, the authors show that ε-Fe_2_C phase can be stabilized even at ~ 573 K by being encapsulated inside graphene layers, and retains high activity in FTS.

## Introduction

Fischer–Tropsch (FT) synthesis transforms syngas (a mixture of CO and H_2_) into multi-carbon hydrocarbons, which can be liquid fuels and chemicals. Because FT synthesis is a core reaction in the utilization of various non-petroleum carbon sources (such as coal, natural or shale gas, biomass, and CO_2_) to supply energy and chemicals, the development of efficient FT catalysts has received much-renewed interest in recent years^1–6^. Iron-based catalysts have widely been used in the industrial FT process because of the low cost of iron, wide operation conditions, and flexible product distributions^7^. However, Fe-based catalysts usually suffer from low activity and stability^3^, and thus many recent fundamental studies have been devoted to enhancing the FT activity and stability of Fe catalysts by employing different modifiers or different supports^8–14^. Unlike Ru- or Co-based FT catalysts, where metallic Ru^0^ or Co^0^ functions as the active phase, metallic Fe^0^ is unstable and the evolution of a conventional Fe-based catalyst typically results in a mixture of different iron phases including Fe_3_O_4_ and iron carbides under FT reaction conditions^15–21^. Iron carbides are believed to be responsible for the activation of CO and the chain growth in FT synthesis, but the nature of the true active iron-carbide phase is still under debate and this hinders the rational design of highly active and stable Fe-based FT catalysts.

**Fig. 1:**
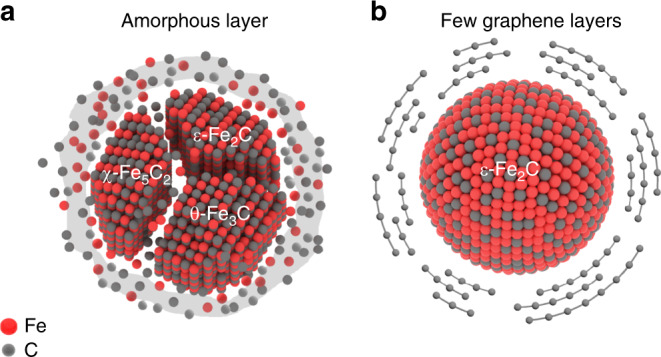
Schematic models of iron-based catalysts for Fischer–Tropsch synthesis. **a** Conventional catalysts with unconfined iron carbide (Fe_*x*_C) particles as the active phase. **b** Graphene layer-confined ε-Fe_2_C.

Hägg χ-Fe_5_C_2_ has been observed in many Fe-based catalysts after FT reactions or during in situ characterizations^22–24^. These observations form the current consensus that χ-Fe_5_C_2_ is the active phase for FT synthesis. The pure-phase χ-Fe_5_C_2_ was also successfully synthesized and was confirmed to be efficient for FT synthesis^25,26^. Theoretical calculations also predicted that χ-Fe_5_C_2_ surfaces catalyzed the CO activation and C-C chain growth^27,28^, and χ-Fe_5_C_2_ should be more active than metallic Fe^27^. Nevertheless, a recent work disclosed that the octahedral carbide ε-Fe_2_C, which contains carbon atoms in octahedral interstices of hexagonal closed-packed iron lattice, was more active than a χ-Fe_5_C_2_-dominant catalyst in the low-temperature (≤473 K) FT reaction^29^. The Fe catalyst based on ε-Fe_2_C phase could also decrease the CO_2_ selectivity during FT synthesis^30^. However, it is known that ε-Fe_2_C would be transformed into χ-Fe_5_C_2_ at above 523 K, and thus would be unstable at a higher temperature (~573 K) that is usually adopted for Fe-catalyzed FT synthesis. Under FT reaction, Fe-based catalysts are usually coated with an amorphous carbon/carbide layer that facilely induces the carbides transformation (Fig. 1a)^31,32^. Thus, it is highly challenging to synthesize stable catalysts that are dominated by the highly active ε-Fe_2_C phase for FT synthesis.

Here, we attempt to replace the amorphous carbon with few graphene layers that confines the Fe-based catalyst. The confinement of the rigid geometry of the graphene shell can inhibit the formation of the amorphous carbon layer and improve the stability of highly active ε-Fe_2_C (Fig. 1b). We report our finding that the confinement of ε-Fe_2_C inside graphene layers (denoted as ε-Fe_2_C@graphene) can stabilize this metastable phase for FT synthesis at 523–613 K. The Fe-time yield (FTY), which is defined as the moles of CO converted to hydrocarbons per gram of Fe per second, reaches 1258 μmol_CO_ g_Fe_^−1^ s^−1^ at 613 K, breaking the upper-limit value (1000 μmol_CO_ g_Fe_^−1^ s^−1^) reported for Fe-based FT catalysts^12,13^. The catalyst is highly stable under our FT reaction conditions and high CO conversion (~ 95%) can be kept at 573 K at least for 400 h. DFT calculations suggest that the confinement effects of graphene layers favor the formation of ε-Fe_2_C from carburization of α-Fe, which maintains the high stability of ε-Fe_2_C under high-temperature FT reaction conditions. On the other hand, the facile transformation of Fe_*x*_C particles may occur during FT synthesis over the conventional FT catalyst. To the best of our knowledge, this is the first example to demonstrate experimentally that the ε-Fe_2_C phase can be stabilized under high-temperature FT reaction conditions. The present work provides a promising strategy to synthesize highly active and stable Fe-based FT catalysts and offers an opportunity for the study of FT reactions on pure metastable-phase iron carbides.

## Results

### Catalysts structure and structure evolution

**Fig. 2:**
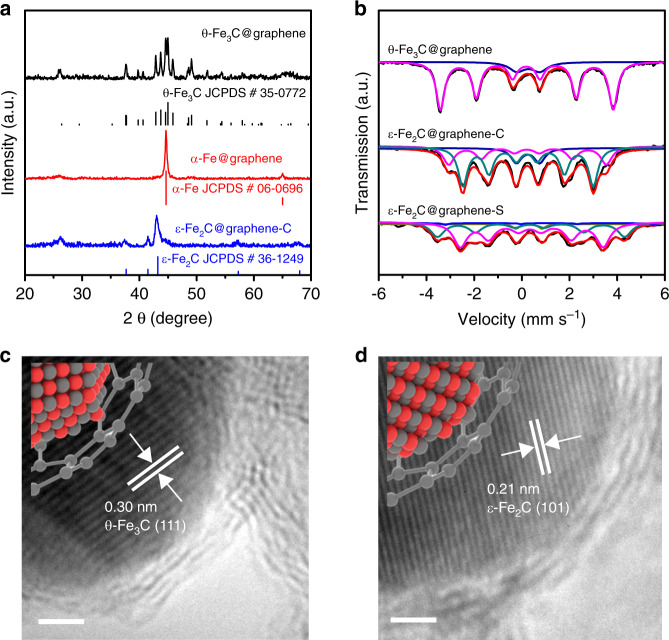
Structure of θ-Fe_3_C@graphene and ε-Fe_2_C@graphene samples. **a** XRD diffraction patterns for θ-Fe_3_C@graphene sample (black line), θ-Fe_3_C@graphene sample reduction under flowing H_2_ at 623 K for 3 h (red line), and then carbonization under flowing syngas (H_2_/CO = 1) at 573 K for 10 h (blue line). **b** ^57^Fe Mössbauer spectra for θ-Fe_3_C@graphene, ε-Fe_2_C@graphene-C, and ε-Fe_2_C@graphene-S samples. Representative high-resolution TEM micrographs for **c** θ-Fe_3_C@graphene and **d** ε-Fe_2_C@graphene-C. Scale bar, 2 nm.

Our confined iron carbide catalyst was synthesized by a pyrolysis method, followed by reduction with H_2_ and carburization in syngas flow. X-ray diffraction (XRD) measurements and ^57^Fe Mössbauer spectroscopy showed that θ-Fe_3_C was the major Fe phase in the precursor obtained after pyrolysis (Fig. 2a, b). Transmission electron microscopy (TEM) results clarified that most of the iron carbide nanoparticles were well dispersed with a near-spherical morphology and had a mean size of 13.6 nm (Supplementary Fig. 1a). The high-resolution TEM (HRTEM) studies revealed that the θ-Fe_3_C nanoparticles were surrounded and closely attached by graphene layers (Fig. 2c and Supplementary Fig. 1b). The lattice fringes with *d* spacing values of 0.30 and 0.34 nm, which corresponded to the (111) facet of θ-Fe_3_C and the (002) facet of graphene, respectively, were observed (the insert of Supplementary Fig. 1b). These results indicate that the precursor obtained after pyrolysis is in the structure of θ-Fe_3_C@graphene.

After the reduction by H_2_ at 623 K, α-Fe became the major iron phase, indicating that θ-Fe_3_C was decomposed into Fe (Fig. 2a). This was also confirmed from HRTEM images, showing that the nanoparticle of α-Fe remains encapsulated by graphene layers after the reduction. The carburization of α-Fe@graphene under syngas at 573 K transformed α-Fe into iron carbide again (denoted as ε-Fe_2_C@graphene-C). It is of interest that ε-iron carbide (ε-Fe_2_C) rather than θ-Fe_3_C or χ-Fe_5_C_2_ was formed after the carburization (Fig. 2a). The HRTEM result reveals that the ε-iron carbide is surrounded by the graphene layers (Fig. 2d). It can be clearly seen by spherical aberration-corrected scanning transmission electron microscopes (Cs-corrected STEM) that the graphene layers of ε-Fe_2_C@graphene catalyst are about 2–7 layers and a large number of defects spread over carbon layer outside ε-Fe_2_C@graphene catalyst (Supplementary Fig. 2).

For graphene layers covered metal catalysts, it was demonstrated that defects on graphene layers render the channels for the diffusion of active species through the grain boundaries on the metal surface^33^. Molecules such as CO, H_2_, and H_2_O can go through domain boundaries and point defects (such as pentagon-heptagon defects and vacancies) on the 2D material overlayers, which mainly follow the defect-aided intercalation mechanism^34^. In addition, the Raman spectra of ε-Fe_2_C@graphene catalyst show that the 2D peak position of ε-Fe_2_C@graphene blueshifted relative to single-layer graphene and the peak pattern consistent to the few graphene layers rather than bulk graphite (Supplementary Fig. 3).

^57^Fe Mössbauer spectroscopy of ε-Fe_2_C@graphene-C provided further evidence for the formation of ε-Fe_2_C after the carburization (Fig. 2b and Supplementary Table 1). Moreover, after 100 h reaction in syngas at 573 K (denoted as ε-Fe_2_C@graphene-S), the ε-Fe_2_C content remained constant (about 62.8%), indicating the stabilizing effect by the graphic carbon layer on ε-Fe_2_C even under reaction conditions (Fig. 2b and Supplementary Table 1).

**Fig. 3:**
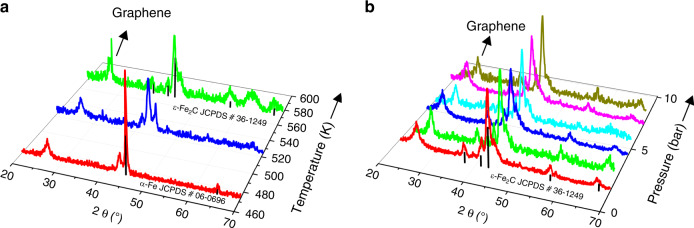
XRD patterns for θ-Fe_3_C@graphene samples. **a** θ-Fe_3_C@graphene samples reduction under flowing H_2_ at 623 K for 3 h, and then treated under syngas (H_2_/CO = 1) at different temperature. **b** θ-Fe_3_C@graphene samples reduction under flowing H_2_ at 623 K for 3 h, and then treated under different CO pressure at 573 K.

To gain further insights into the possible evolution of iron phases, we have performed in situ XRD characterizations for our sample in syngas flow under different conditions. α-Fe was still the major phase under syngas with a H_2_/CO ratio of 1 at 473 K and was gradually changed to ε-Fe_2_C phase upon increasing the temperature from 473 to 573 K (Fig. 3a). In addition, during the carburization process, ε-Fe_2_C phase kept stable in syngas flow at 573 K for 5 h and remained almost unchanged by changing the CO pressure at 573 K (Fig. 3b). It is noteworthy that conventionally ε-Fe_2_C is unstable and would be converted to χ-Fe_5_C_2_ and θ-Fe_3_C at ≥ 523 K^20^. Interestingly, we observed the formation of ε-Fe_2_C phase in a wide range of temperature and CO pressure, which are closely related to the model of carbon chemical potential (*μ*_C_) as explained in the computational details of Supplementary Methods. Thus, the present results clearly demonstrate that the confinement of ε-Fe_2_C inside graphene layers can keep it from phase transformations probably.

For comparison, we further studied the changes in structures of θ-Fe_3_C@graphene samples before and after treatment under different conditions (Supplementary Fig. 4). The direct carburization of θ-Fe_3_C@graphene did not induce any detectable change in the phase composition, probably because this sample was formed by a prior carburization process. Fe_2_O_3_ was obtained by oxidation of θ-Fe_3_C@graphene and served as a carbon encapsulation-free reference. The carburization treatment of Fe_2_O_3_ led to the formation of Fe_3_O_4_ and χ-Fe_5_C_2_. The characterization result shows that only χ-Fe_5_C_2_ could be observed at 573 K, which is the thermodynamic stable phase at high temperatures. On the other hand, the carbon-encapsulated metal Fe sample only formed the ε-Fe_2_C phase after the carburization treatment. The ε-Fe_2_C phase can be stabilized by the graphene layer even at such a high temperature.

Raman spectroscopy was used to identify the nature of the graphene layers. Two bands at 1350 and 1590 cm^−1^ were observed in the Raman spectra for ε-Fe_2_C@graphene-C and ε-Fe_2_C@graphene-S, corresponding to the D-band (disordered carbon) and the G-band (graphene carbon), respectively (Supplementary Fig. 5). A lower *I*_D_/*I*_*G*_ intensity ratio for the ε-Fe_2_C@graphene was observed, consistent with a higher degree of graphene of the carbon matrix^35^. The 2D bands of the two ε-Fe_2_C@graphene samples are closely related to the band structure of graphene layers^36^. These observations suggest that while the iron phase undergoes changes under different atmospheres, the graphene layers keep surrounding the particle of iron or iron carbide.

In addition, the surface composition of the graphene layers is of particular interest and surface contents of N, O, and Fe were determined by X-ray photoelectron spectroscopy (XPS). The θ-Fe_3_C@graphene sample has a surface atomic ration of Fe/C of about 0.03, and the surface contents of N, O, and Fe elements did not vary with an increase of the Fe loading in the catalysts (Supplementary Table 2). The results revealed a negligible surface iron content, suggesting that θ-Fe_3_C nanoparticles were encapsulated by the graphene layers in the catalysts.

XPS characterizations were further performed to analyze carbon and nitrogen bonding configurations in the carburization process of Fe@graphene. N 1s peaks in XPS of θ-Fe_3_C@graphene (Supplementary Fig. 6a, b) can be fitted into four peaks at 398.3 eV, 399.6 eV, 400.8 eV, and 402.5 eV referring to the pyridinic, pyrrolic, graphitic and oxidized nitrogen, respectively^37^. These results confirmed the existence of N functional groups (pyridinic-N, pyrrolic-N, and graphitic-N), indicating unique defect-rich structure graphene layers after the annealing process. Moreover, the XPS results clearly confirmed the presence of defective graphene layers during the reducing and carburization processes (Supplementary Fig. 6c, e) and the incorporation of nitrogen atoms within the graphene layers (Supplementary Fig. 6d, f). From the above Raman spectra, STEM/HRTEM images, and XPS results, we conclude that the highly defective graphene layers have been successfully synthesized during the thermal annealing. The number of defects and type of doped N in carbons might play a crucial role in enhancing FT reaction catalytic performance^38,39^. N 1s XPS spectra of the few graphene layers confined iron catalysts reveals that the graphitic N is the most abundant N species, indicating that the graphitic N could affect the performance of confined iron catalysts, which is rationalized by our theoretical modeling shown in the part of DFT calculation.

### Catalytic performance of ε-Fe_2_C phase in high-temperature FT synthesis

It was once reported that the activity of ε-Fe_2_C nanoparticles is 4.3 times that of χ-Fe_5_C_2_ and is even comparable to that of the precious metal Ru for FT synthesis, probably owing to its excellent ability to dissociate CO^29^. Unfortunately, the study of the catalytic performance of ε-Fe_2_C under practical FT reaction conditions is limited because of its metastable state.

Here, the thermal stability ε-Fe_2_C@graphene enables us to investigate its catalytic performance at 573 K. The FTY value of ε-Fe_2_C@graphene at 573 K was 582.8 μmol_CO_ g_Fe_^−1^ s^−1^ (Table 1), which was significantly higher than those for the un-encapsulated χ-Fe_5_C_2_ derived from Fe_2_O_3_ and the θ-Fe_3_C@graphene catalysts. The intrinsic activity (TOF values) of ε-Fe_2_C is ~6–10 times higher than the θ-Fe_3_C and 2 times higher than the χ-Fe_5_C_2_. Furthermore, CO-TPD profiles of ε-Fe_2_C@graphene catalyst show a multi-peak overlapped cure with a maximum peak position at *ca*. 843 K, which is attributed to desorption of CO after recombination of dissociated carbon and oxygen on the surface (Supplementary Fig. 7). The result revealed the strongly bound CO on the surface due to the confinement effect of ε-Fe_2_C@graphene catalyst. In addition, the CO_2_ selectivity for the ε-Fe_2_C@graphene catalyst was lower than those for the other two reference catalysts, indicating that the ε-Fe_2_C is a more active and selective phase for the conversion of syngas to hydrocarbons.

**Table 1:** Activity and hydrocarbon selectivity of different iron carbides catalysts for Fischer–Tropsch synthesis^a^.

Catalyst	FTY (µmol_CO_ g_Fe_^−1^ s^−1^)	CO_2_ sel. (%)	O/P ratio (C_2_-C_4_)	CH_4_ sel. (%)	C_2_-C_4_ sel. (%)	C_5_^+^ sel. (%)	TOF ^c^ (s^−1^)×10^2^	TOF ^d^ (s^−1^)×10^2^
χ-Fe_5_C_2_^b^	62.5	44.6	1.3	11.5	30.0	58.5	4.7	5.6
θ-Fe_3_C@graphene	35.7	39.9	2.4	15.3	45.0	39.7	1.6	1.1
ε-Fe_2_C@graphene	582.8	20.3	2.6	10.3	23.9	65.8	10.2	11.4

^a^Reaction conditions: H_2_/CO = 1/1, 573 K, *p* = 10 bar.

^b^χ-Fe_5_C_2_ was obtained from the θ-Fe_3_C@graphene sample by oxidation at 723 K for 5 h in air, and then carbonization under flowing syngas (H_2_/CO = 1) at 573 K for 10 h (the sample without carbon encapsulation denoted as χ-Fe_5_C_2_).

^c^Based on CO chemisorption.

^d^Based on iron carbide particle size^41^, and calculated by using the densities of ε-Fe_2_C, χ-Fe_5_C_2_, and θ-Fe_3_C of 7.19 g mL^−1^, 7.57 g mL^−1^, and 7.68 g mL^−1^ respectively, and by assuming 14 Fe atoms nm^−^^2^.

**Fig. 4:**
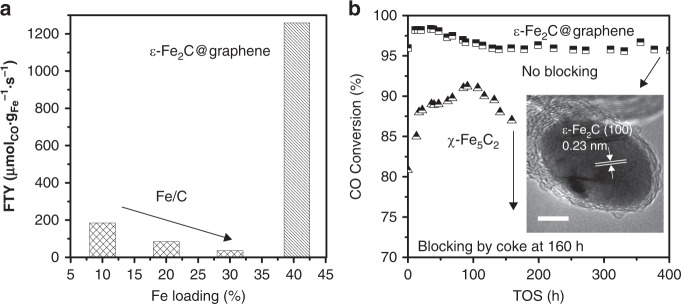
Catalytic performance of different iron catalysts. **a** Comparison of FTY values between ε-Fe_2_C@graphene catalyst and iron catalyst loaded on active carbon with different iron loadings. **b** Long-term stability of ε-Fe_2_C@graphene and un-encapsulated χ-Fe_5_C_2_ catalysts. Reaction conditions: H_2_/CO = 1/1, 573 K, *p* = 10 bar. The insert shows the high-resolution TEM micrograph for the spent ε-Fe_2_C@graphene catalysts after 400 h reaction. Scale bar, 5 nm.

The ε-Fe_2_C@graphene catalysts with different Fe loadings all showed very high activities. The FTY value for the ε-Fe_2_C@graphene catalysts with Fe loadings in a range of 10–50 wt% is almost the same (~ 600 µmol_CO_ g_Fe_^−1^ s^−1^) under the same reaction condition. On the other hand, the FTY value of reference Fe/C catalysts decreased sharply upon increasing Fe loading probably due to the aggregation of Fe species and the oxidation of the active carbide phase under reaction conditions (Fig. 4a)^40,41^. The keeping of high FTY value at high Fe loading suggests that the high dispersion of ε-Fe_2_C phase in the ε-Fe_2_C@graphene catalyst is sustained at high Fe loadings and the ε-Fe_2_C keeps stable during FT reaction. The FTY value of ε-Fe_2_C@graphene with iron loading of 40.5 wt% reached 1258 μmol_CO_ g_Fe_^−1^ s^−1^ when the gas-hour space velocity (GHSV) was increased to 160 L g_cat_^−1^ h^−1^ at 613 K (Fig. 4a). The FTY values reported to date are limited at 1000 μmol_CO_ g_Fe_^−1^ s^−1^ (Supplementary Table 3) and the FTY value obtained using the ε-Fe_2_C@graphene catalyst breaks this limitation.

Time-on-steam evolution of CO conversion for ε-Fe_2_C and χ-Fe_5_C_2_ at the same conversion (~ 50%) with different GHSVs revealed that the ε-Fe_2_C@graphene is much more active than χ-Fe_5_C_2_ in FT synthesis, as shown in Supplementary Fig. 8. It was also found that the carburization of Fe@graphene into ε-Fe_2_C@graphene was completed during 50 h at GHSV of 64.0 L g_cat_^−1^ h^−1^. For evaluation of catalyst deactivation, we have performed long-termed FT reactions for both ε-Fe_2_C@graphene and un-encapsulated χ-Fe_5_C_2_ catalysts at 573 K under harsh conditions (Fig. 4b). The CO conversion underwent a significant decrease when the time-on-steam exceeded 100 h for the un-encapsulated χ-Fe_5_C_2_ catalyst and this catalyst was covered by carbon. The pressure drop across the un-encapsulated χ-Fe_5_C_2_ catalyst bed increased after 100 h and the gas flow was totally blocked at 160 h, as shown in Fig. 4b. On the other hand, the encapsulated ε-Fe_2_C sample was stable for more than 400 h even at a higher CO conversion (~ 95%). Further, the pressure drop across the ε-Fe_2_C@graphene catalyst bed was negligible even at high conversion, indicating that the coke deposition on this catalyst was significantly suppressed by the graphene layers under harsh condition.

Furthermore, the selectivity toward olefins and long-chain hydrocarbons also did not undergo significant changes during the long-term reaction. The hydrocarbon distribution over the ε-Fe_2_C@graphene catalyst follows the Anderson-Schulz-Flory distribution (Supplementary Fig. 9). Our Raman studies for the catalyst after reaction showed that the graphene layers and ε-Fe_2_C phase did not undergo significant changes (Supplementary Fig. 5). The morphology and crystalline structure of the ε-Fe_2_C@graphene catalyst also kept almost unchanged after 400 h reaction.

### DFT calculation of graphene confinement on ε-Fe_2_C

**Fig. 5:**
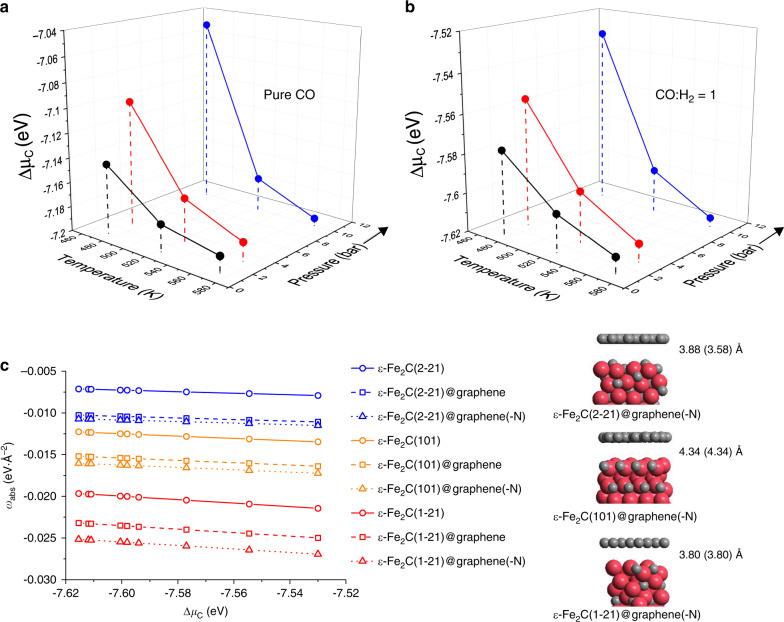
DFT calculation of graphene confinement on ε-Fe_2_C. **a** Relative chemical potential of carbon (Δ*μ*_C_) for carburization by CO (2CO → C + CO_2_). **b** Relative chemical potential of carbon (Δ*μ*_C_) for carburization by syngas (4CO + 4H_2_ → 2C + CO_2_ + 2H_2_O + CH_4_). **c** Surface-normalized carbon absorption energy (*ω*_abs_) of ε-Fe_2_C surfaces with and without graphene(-N) layers and the most stable structures labeled by the distances between ε-Fe_2_C and graphene (data in parenthesis referring to those of graphene-N).

The effects of a graphene layer on the surface stability and carburization feasibility of ε-Fe_2_C phases were investigated by ab initio atomic thermodynamics based on DFT calculations. The relative chemical potential of carbon (Δ*μ*_C_) is relevant in describing the thermodynamics of iron carbides, which can be determined from the equilibrium of carburization reactions of different gas atmospheres for pretreatment or FT reaction at some temperatures and pressures. Higher temperature and lower pressure, as well as higher H_2_/CO ratio, result in lower Δ*μ*_C_ value indicating a lower carburization ability (Fig. 5a, b). Based on Δ*μ*_C_, the carbon absorption energy (*E*_abs_) can be derived for describing the carburization reaction from metallic Fe to iron carbide. The volume-normalized carbon absorption energy (*Ω*_abs_) was applied to the bulk iron carbides^20^.

Here, we develop a surface-normalized carbon absorption energy (*ω*_abs_), which is suitable for describing the surface carburization of iron carbide with and without graphene confinement. The most abundant (101), (1-21), and (2-21) surfaces were selected to evaluate the confinement effects of graphene layers on the *ω*_abs_ (Fig. 5c). The negative *ω*_abs_ values indicate the favorable stability of the ε-Fe_2_C surfaces under the conditions of pretreatment and FT reaction. Although the surface stability follows the order of (101) > (1-21) > (2-21) according to the calculated surface energy (Supplementary Table 4), the (1-21) has the highest carburization feasibility from metallic iron because of the lowest *ω*_abs_ value.

On the other hand, the confinement effects are modeled by covering the ε-Fe_2_C surfaces with a single graphene layer. The optimized distance between ε-Fe_2_C and graphene is *ca*. 3.9–4.3 Å (Fig. 5c) close to the dynamic diameter of reactants and single-chain hydrocarbons^42,43^, which can afford the catalytic FT reaction on the ε-Fe_2_C surfaces with the graphene confinements. The *ω*_abs_ values for the graphene-covered ε-Fe_2_C are lower than those of the pristine ε-Fe_2_C surfaces, indicating the improved thermodynamic stability. The N-doped graphene (graphene-N) show similar results to those of graphene.

In addition, taking (1-21) as an example, we further investigate the mechanism of CO dissociation on the ε-Fe_2_C surface with and without graphene confinement. Both the direct and H-assisted dissociations are considered including different active sites of perfect ε-Fe_2_C, graphene, and the C-vacancy on ε-Fe_2_C (Supplementary Figs. 11–15). The C-vacancy on ε-Fe_2_C is more active for the direct CO dissociation with a lower energy barrier (*E*_a_) of *ca*. 1.2 eV than those of direct and H-assisted dissociations (2.57-2.61 and 2.83-2.87 eV) on the perfect ε-Fe_2_C surface with and without the confinement of graphene. The CO dissociation on the graphene site hardly occurs due to the high *E*_a_ values (> 2.89 eV). Similar results can be found for graphene-N. The confinement of graphene or graphene(-N) favors to improve the stability of the highly active ε-Fe_2_C to achieve the high catalytic performance of FT at high temperature.

## Discussion

In summary, we synthesized graphene layers encapsulated ε-Fe_2_C nanocrystals for the FT reaction. It exhibited remarkably activity (~ 1258 μmol_CO_ g_Fe_^−1^ s^−1^) and stability (>400 h) under realistic FT synthesis conditions. The confinement effects of graphene layers stabilize the metastable but very active ε-Fe_2_C phase. The unique confinement structure (ε-Fe_2_C@graphene) can inhibit the formation of an amorphous carbon layer that converts catalytically active ε-Fe_2_C phase to other less active carbide phases (e.g., χ-Fe_5_C_2_). Our results and conclusion could help in the rational design of promising active phases in industrial catalysts for hydrogenation processes.

## Methods

### Preparation of θ-Fe_3_C@graphene

The iron carbide nanocomposites were synthesized by pyrolysis of a molten mixture of urea, glucose, and Fe(NO_3_)_3_•9H_2_O. In a typical synthesis, an amount of Fe(NO_3_)_3_•9H_2_O, corresponding to the final Fe loading of 30–60 wt%, was mixed with 5.0 g urea and 3.0 g glucose at 393–433 K to form a transparent solution. The resultant molten mixture was heated at 453 K in an oven for 24 h. The solid collected was subjected to a heat treatment in flowing N_2_ (10 mL min^−1^) at 673 K for 30 min and finally at the final temperatures (773–1023 K) for another 2 h.

### Preparation of ε-Fe_2_C@graphene

The θ-Fe_3_C@graphene samples obtained above were reduced in a flow of 3 L g_cat_^−1 ^h^−1^ of H_2_ at 623 K for 3 h (denoted as Fe@graphene), and then the carbonization in a flow of 64 L g_cat_^−1^ h^−1^ of syngas (H_2_/CO = 1) at 573 K for 10~400 h (denoted as ε-Fe_2_C@graphene).

### Catalyst Characterization

The size and morphology of samples were determined using a FEI Tecnai G20 transmission electron microscope operated at 200 kV and a Hitachi SU8000 field emission scanning electron microscope at an accelerating voltage of 15 kV.

The ^57^Fe Mössbauer measurements were performed at room temperature or 77 K using a conventional spectrometer (Germany, Wissel MS-500) in transmission geometry with constant acceleration mode. A 57 Co(Rh) source with an activity of 25 mCi was used. The velocity calibration was done with a room temperature α-Fe absorber. The spectra were fitted by the software Recoil using Lorentzian Multiplet Analysis. The samples were passivated in flowing 1% O_2_/N_2_ for 1 h at room temperature prior to air exposure and being sealed in a sample holder with paraffin wax for Mössbauer spectroscopy measurements.

The CO chemisorption was performed using a Micromeritics AutoChem II 2920 unit. Before CO chemisorption, 0.1 g of catalyst was reduced under flowing pure hydrogen at 623 K for 3 h and then carburized in syngas (H_2_/CO = 1) at 573 K for 5 h. Subsequently, the adsorbed species were removed by flowing He at 823 K for 2 h. The samples were cooled to 308 K, then CO chemisorption experiment was conducted. The average CO:Fe stoichiometry was assumed 1:2. For θ-Fe_3_C@graphene catalyst, reduction and carburization processes are eliminated.

For CO temperature-programmed desorption (CO-TPD), 0.1 g of catalyst was reduced under flowing pure hydrogen at 623 K for 3 h and then carburized in syngas (H_2_/CO = 1) at 573 K for 5 h. Subsequently, the adsorbed species were removed by flowing He at 573 K for 2 h. The samples were cooled to 308 K. At this temperature, the carburized sample was flushed with CO for 1 h and consequently purged with He until the baseline of CO signal leveled off. Finally, the sample was heated to 1,073 K at a ramp of 10 K min^−1^. For θ-Fe_3_C@graphene catalyst, reduction and carburization processes are eliminated.

X-ray diffraction (XRD) patterns of the samples were recorded on a Bruker D8 powder diffractometer equipped with a Cu-Kα source operated at 40 kV and 40 mA and a Vantec-1 detector.

X-ray photoelectron spectroscopy (XPS) measurements were conducted on a VG Multilab 2000 photoelectron spectrometer using Al *K*_α_ radiation operated under vacuum (2 × 10^−6^ Pa) with the binding energy (BE) calibrated using the C 1s peak at 284.6 eV.

Thermogravimetric analysis (TGA) was carried out on a NETZSCH TG 209F3 TGA analyzer during temperature ramping from 303 K to 1,173 K in flowing air (50 mL min^−1^) with a ramping rate of 10 K min^−1^.

### Catalytic evaluation

Fischer–Tropsch synthesis was performed in a stainless steel fixed-bed reactor (i.d. = 12 mm). Catalysts were diluted with inert SiC particles in a mass ratio of 1:10 prior to testing. The diluted catalysts (5.5 g) were pre-treated in flowing H_2_ (10 mL min^−1^) at 623 K for 3 h before reactions. The pre-treated catalysts were cooled to 373 K in flowing H_2_ before the introduction of syngas (H_2_/CO = 1). The reaction temperature was then ramped slowly to 593 K. The permanent gases (H_2_, CO, CO_2_) and light alkanes (CH_4_, C_2_H_6_, etc.) in the effluent of the reactor were monitored by an online Agilent Micro GC3000A gas chromatograph (GC) equipped with the molecular sieve, Plot-Q and Al_2_O_3_ capillary columns and a TCD detector. The oil and wax products were separated using a cold trap (271 K) and a hot trap (423 K), respectively, while the aqueous products were obtained by phase separation in those traps. The oil products were analyzed using an Agilent 6890N GC with a FID detector and a HP-5 column; the wax products were dissolved in CS_2_ and analyzed using an Agilent 7890A GC equipped with a FID detector and a HT5 column; the aqueous fraction was analyzed using an Agilent 4890 GC equipped with a FID detector and a HP-Innowax column. The product selectivity was calculated based on the carbon balance.

### DFT calculations

Density functional theory calculations were performed using the generalized gradient approximation Perdew–Burke– Ernzerhof of (PBE) functional^44^ and projector-augmented wave (PAW) method^45^ as implemented in the Vienna ab initio simulation package (VASP)^46^. A second-order Methfessel–Paxton electron smearing scheme (sigma = 0.2 eV) was used because of the metallic conductor properties of iron carbide. Plane-wave kinetic energy cut off of 400 eV is sufficiently accurate for the spin polarization calculations of the electronic properties of open-shell iron carbide. Energy and force convergence criteria were 10^−5^ eV and 0.03 eV Å^−1^, respectively. The most abundant ε-Fe_2_C(1-21), ε-Fe_2_C(101), and ε-Fe_2_C(2-21) surfaces were taken into account. A vacuum of 15 Å was used for screening the interactions vertical to the surface. The most stable configurations were selected to model the confinement effects of graphene and N-doped graphene (graphene-N) on the carburization feasibility of ε-Fe_2_C from metallic iron and the reaction mechanism of CO dissociation. The computational details are deposited in Supplementary Methods.

## Supplementary information

Supplementary Information

## Data Availability

The data that support the plots within this paper and other findings of this study are available from the corresponding author upon request.
