# Supplementary material for: Stabilization of ε-iron carbide as high-temperature catalyst under realistic Fischer–Tropsch synthesis conditions
Source: Nat Commun. 2020 Dec 4;11:6219. doi: 10.1038/s41467-020-20068-5 (PMC7719174; doi:10.1038/s41467-020-20068-5)
Supplement: Supplementary file 1 — Supplementary Information [file 41467_2020_20068_MOESM1_ESM.pdf]

Supplementary Information

Stabilization of ε-iron carbide as high-temperature catalyst under realistic Fischer-Tropsch synthesis conditions

Lyu et al.

Supplementary Methods

Computational details

Bulk structure of ε-Fe_2C was optimized with constraint of the experimental symmetry (*P*63/*mmc*). Both total energy and lattice structures were converged to no more than 1 meV and 1 pm, respectively, with increasing the Monkhorst-Pack grid of *k*-point. A supercell of $p(2 \times 2 \times 2)$ was applied for the ε-Fe_2C taking the most stable configuration as reported in the literature[1]. The optimized bulk structure of ε-Fe_2C was truncated into $p(2 \times 2 \times 1)$, which was used for cutting the surface model of the most abundant (1-21), (2-21) and (101) with a slab thickness of no less than 10 Å and a vacuum of 15 Å, in which all atoms were fully relaxed. Various terminations, slab thickness were tested to find the most stable configurations for evaluation of reliable surface energy with a suitable Monkhorst-Pack *k*-point scheme (Supplementary Table 4). A single layer of graphene and N-doped graphene (graphene-N) was applied on the Fe_2C surfaces modeling the confinement effect. The mismatch between the Fe_2C supercells and graphene(-N) layers are no more than 12% (Supplementary Table 5). The optimized distances between Fe_2C and graphene(-N) are determined in the range of 3.58-4.34 Å with low interaction energies (E_{int} < 0.3 eV) (Supplementary Fig. 10).

The chemical potential of C (μ_C) can be defined by the carbon deposition reactions

$$2CO \rightarrow C + CO_2 \quad (1)$$

$$CO + H_2 \rightarrow C + H_2O \quad (2)$$

Thus, μ_C can be written as

$$\mu_C = 2\mu_{CO} - \mu_{CO2} \quad (3)$$

$$\mu_C = \mu_{CO} + \mu_{H2} - \mu_{H2O} \quad (4)$$

in which the chemical potential of CO, CO_2, H_2, and H_2O in ideal gas phase were calculated according to

$$\begin{aligned} \mu &= E_0 + pV - TS \\ &= E_0 + NRT - TS \\ &= E_0 + (NR - S)T \quad (5) \end{aligned}$$

The E_0 is the total energy corrected by the zero-point vibrational energy obtained by frequency calculation using VASP[2]. *N* is 5/2 and 6/2 for linear and nonlinear molecules, respectively. The

entropy (S) at different temperatures and pressures was calculated at PBE/6-31G(d) level as implemented in Gaussian 09 program of packages[3]. Then, $\Delta\mu_C$ is the chemical potential of carbon as referenced to the total energy of a carbon atom.

The CH_4 was also taken into account because the methanation reaction occurs in the syngas pretreatment condition.

$$CO + 3H_2 \rightarrow CH_4 + H_2O \tag{6}$$

The compositions of the gas mixture at equilibrium were determined using NASA chemical equilibrium application code[4].

The Fe_2C surface energy (γ) can be evaluated as follows.

$$\gamma = \frac{E_{\mathrm{slab}}^{\mathrm{Fe_2C}} - \frac{N_{\mathrm{Fe}}}{2} E_{\mathrm{bulk}}^{\mathrm{Fe_2C}} + \left(\frac{N_{\mathrm{Fe}}}{2} - N_{\mathrm{C}}\right)\mu_{\mathrm{C}}}{2A_{\mathrm{slab}}^{\mathrm{Fe_2C}}} \tag{7}$$

in which $E_{\mathrm{slab}}^{\mathrm{Fe_2C}}$ and $E_{\mathrm{bulk}}^{\mathrm{Fe_2C}}$ are the total energies of the slab and bulk Fe_2C, respectively; N_{Fe} and N_C are the numbers of Fe and C in the Fe_2C slab, respectively; $A_{\mathrm{slab}}^{\mathrm{Fe_2C}}$ is the area of the Fe_2C slab.

According to the previous studies, the absorption energy of carbon (E_{abs}) was used for describing the stability and carbonization feasibility of different iron carbide (Fe_xC_y) phases based on calculations on bulk model[5].

$$E_{\mathrm{abs}} = \frac{E_{\mathrm{bulk}}^{\mathrm{Fe}_x\mathrm{C}_y} - xE_{\mathrm{bulk}}^{\alpha\text{-Fe}} - y\mu_{\mathrm{C}}}{y} \tag{8}$$

Here, we develop the concept of surface-normalized carbon absorption energy (ω_{abs}) based on slab model to describe the feasibility of carbonization of the carbide surfaces with and without graphene(-N) confinement ($Fe_xC_yN_z$).

$$\omega_{\mathrm{abs}} = \frac{E_{\mathrm{slab}}^{\mathrm{Fe}_x\mathrm{C}_y\mathrm{N}_z} - xE_{\mathrm{bulk}}^{\alpha\text{-Fe}} - y\mu_{\mathrm{C}} - zE_{\mathrm{N}}}{yA_{\mathrm{slab}}^{\mathrm{Fe}_x\mathrm{C}_y\mathrm{N}_z}} \tag{9}$$

Comparing the ω_{abs} values of the bare and graphene(-N)-covered Fe_2C surfaces renders the effects of graphene carbon on the stability of different Fe_2C surfaces.

In order to define the active sites of the Fe_2C catalyst confined by graphene(-N), we carried out computational study of the reaction mechanisms of the dissociations of carbon monoxide (CO) on the Fe_2C surface with and without graphene(-N) layers. The most stable stoichiometric (1-21)-$p(1 \times 1)$ with five C-Fe-C layers was used as an example in which the bottom three layers were fixed and the top two layers were allowed to relax as well as the graphene(-N) layer and the

CO/H reactants. The structures of initial state (IS), intermediate (IM) and final state (FS) were optimized by the conjugate gradient minimizing algorithm. The transition state (TS) was located by combining the climbing image-nudge elastic band (CI-NEB) and quasi-Newton minimizing techniques, which are verified by frequency calculations (IBRION = 5; POTIM = 0.02 Å). Both direct and H-assisted pathways are considered for those on bare Fe_2C, confined Fe_2C (Fe_2C@graphene and Fe_2C@graphene-N) and supported graphene(-N) sites (graphene/Fe_2C and graphene-N/Fe_2C). The reaction energy profiles and structures are shown in Supplementary Figs. 11-15.

Supplementary Figures and Tables

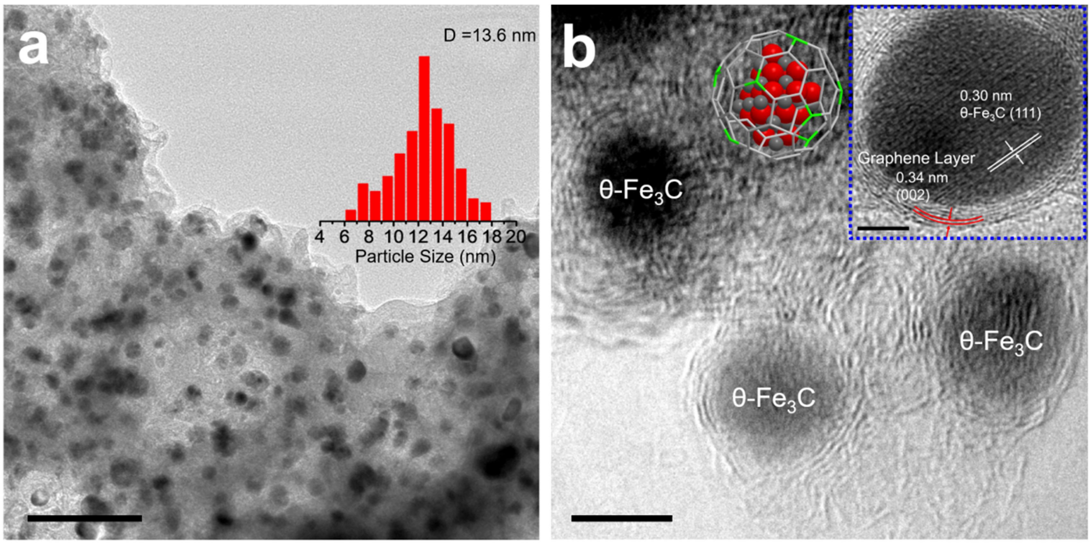

Supplementary Figure 1 TEM images with particle size distribution. **a,** TEM image of θ-Fe_3C@graphene nanocomposites (the insert shows particle size distribution for θ-Fe_3C nanocrystals). Scale bar, 100 nm. **b,** HRTEM image of several θ-Fe_3C@graphene nanocomposites (the insert shows HRTEM image of the interface between a θ-Fe_3C nanocrystal and the graphene layers). Scale bar, 5 nm.

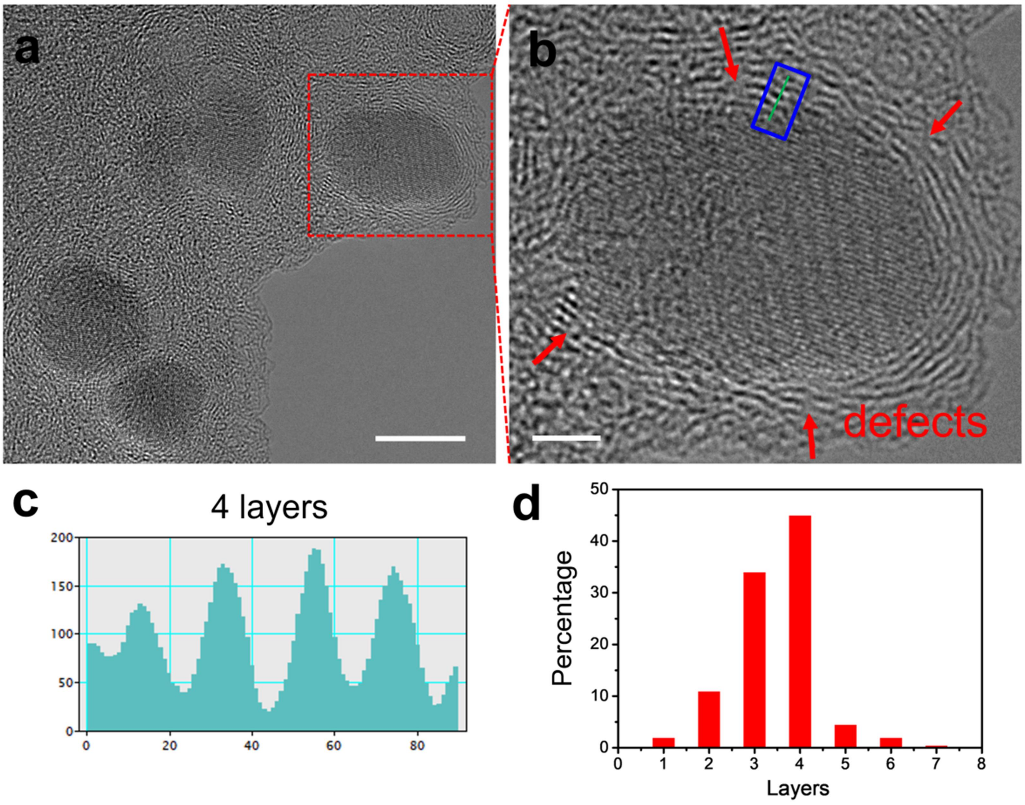

Supplementary Figure 2 Characterizations of ε-Fe_2C@graphene catalyst. **a,** Cs-corrected STEM image of ε-Fe_2C@graphene catalyst. Scale bar, 10 nm. **b,** The magnified image of single particle in a. Scale bar, 2 nm. **c,** Profile of the carbon layers in selected areas in b. **d,** Statistical analysis of the number of layers in the graphene shells encapsulating the iron carbide in ε-Fe_2C@graphene catalyst.

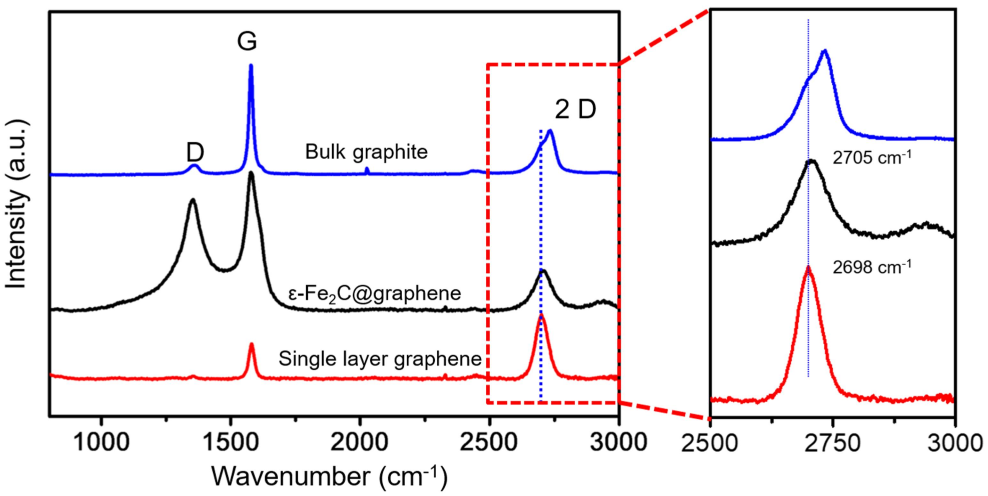

Supplementary Figure 3 Raman spectra of single layer graphene, ε-Fe_2C@ graphene and bulk graphite.

Supplementary Table 1 ^{57}Fe Mössbauer fitted parameters of the as-synthesized iron carbides samples[a]

Samples [b]	QS/mms^{-1}	IS/mms^{-1}	Hyperfine field/kOe	A/%	Phase ascription
θ-Fe_3C@graphene	0.95	0.27	--	15.5	Fe^{2+}/Fe^{3+}
	0.01	0.18	205.7	84.5	θ-Fe_3C
ε-Fe_2C@graphene-C	0.97	0.26	--	12.1	Fe^{2+}/Fe^{3+}
	0.01	0.23	203	27.0	χ-Fe_5C_2
	0.02	0.22	169.7	60.9	ε-Fe_2C
ε-Fe_2C@graphene-S	1.10	0.29	--	2.7	Fe^{2+}/Fe^{3+}
	-0.37	0.77	466.3	2.1	Fe_3O_4
	0.03	0.35	243.6	32.4	χ-Fe_5C_2
	0.04	0.37	185.8	62.8	ε-Fe_2C

[a] Definitions: IS, isomer shift (relative to α-Fe); QS, quadrupole splitting; spectral contribution, relative spectral area.

[b] ^{57}Fe Mössbauer measurements were performed at room temperature (θ-Fe_3C@graphene, ε-Fe_2C@graphene-C) or 77 K (ε-Fe_2C@graphene-S).

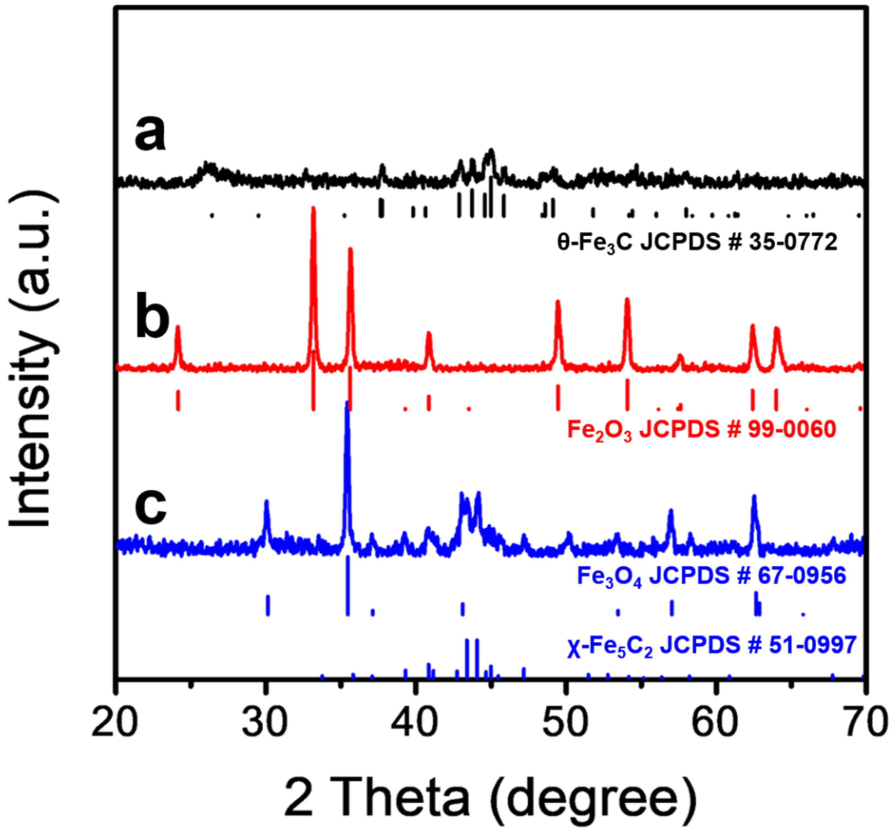

Supplementary Figure 4 XRD diffraction patterns for θ-Fe_3C@graphene sample with different treatment. **a,** as-prepared θ-Fe_3C@graphene sample carbonization under flowing syngas (H_2/CO = 1) at 573 K for 10 h. **b,** as-prepared θ-Fe_3C@graphene sample oxidation at 723 K for 5 h in air, **c,** and then carbonization under flowing syngas (H_2/CO = 1) at 573 K for 10 h.

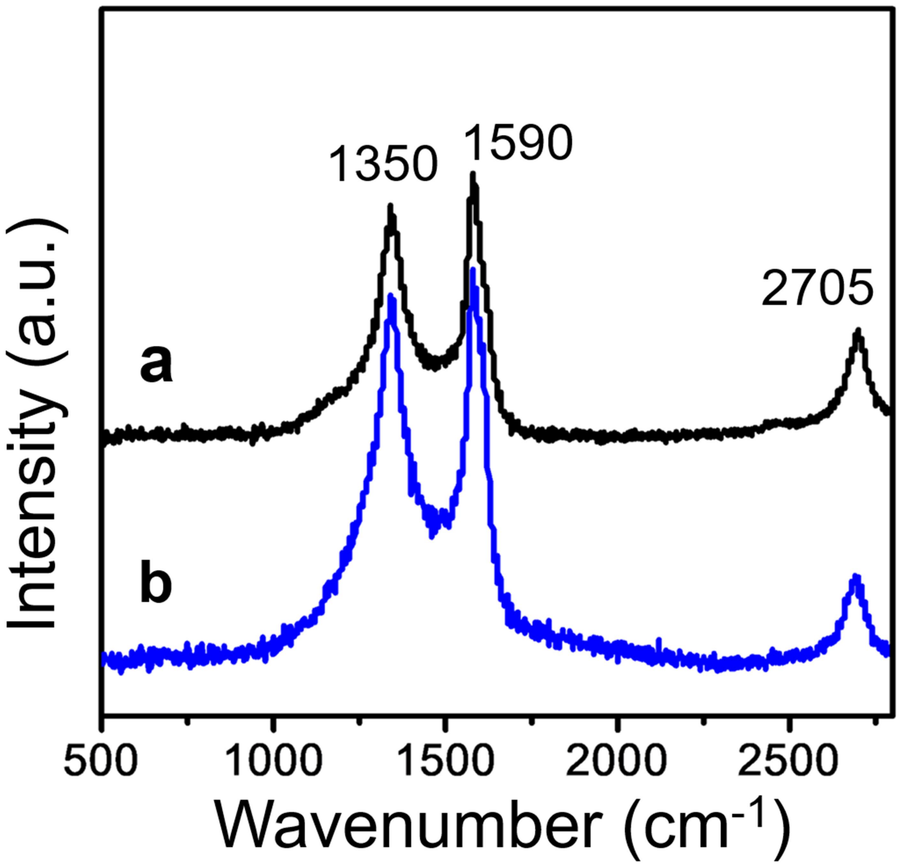

Supplementary Figure 5 Raman spectra of different ε-Fe_2C@graphene samples. **a**, ε-Fe_2C@graphene-C. **b**, ε-Fe_2C@graphene-S.

Supplementary Table 2 Elemental surface compositions of θ-Fe_3C@graphene samples with different iron loading by X-ray photoelectron spectroscopy

Sample [a]	Carbon % (atom. %)	Iron % (atom. %)	Oxygen % (atom. %)	Nitrogen % (atom. %)
25-Fe_3C@graphene	81.8	2.4	10.3	5.5
35-Fe_3C@graphene	85.6	1.8	7.2	5.4
45-Fe_3C@graphene	86.7	1.7	6.8	4.8
55-Fe_3C@graphene	86.7	3.8	7.1	2.4

[a] Label number indicate the weight% Fe in the sample.

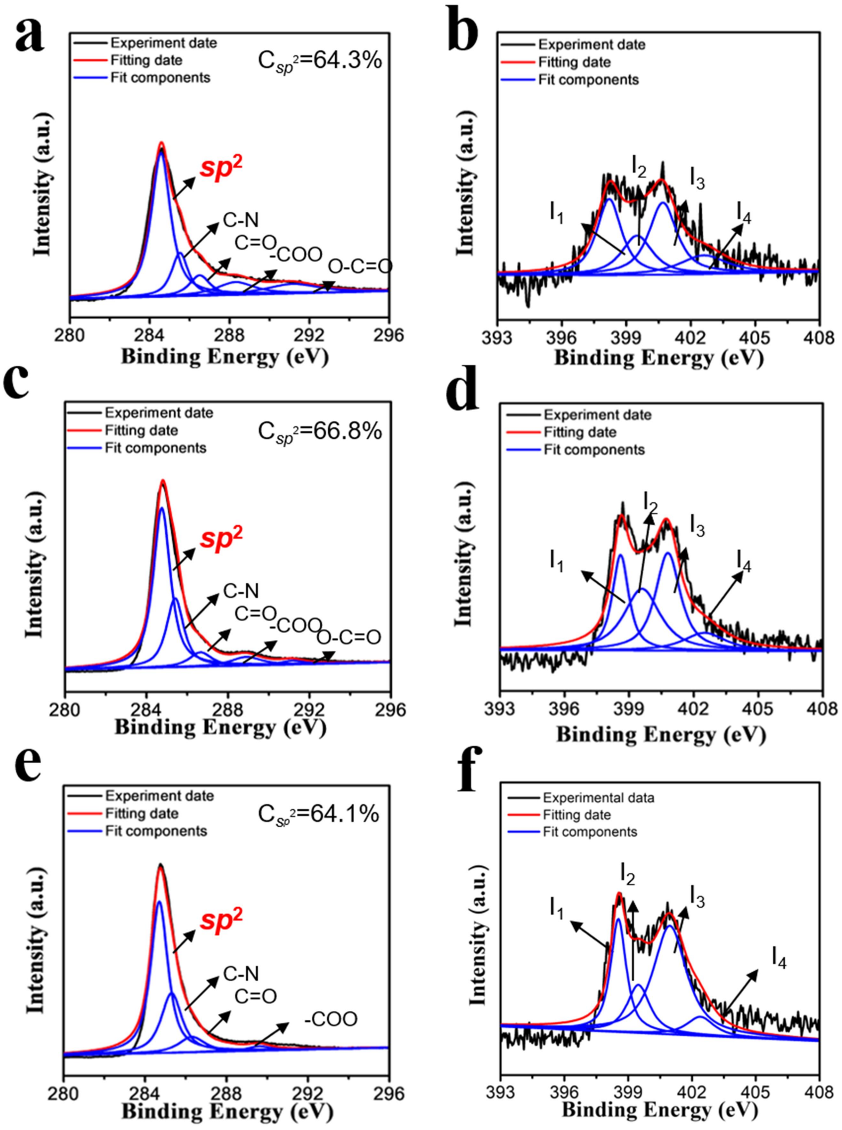

Supplementary Figure 6 XPS spectra for θ-Fe_3C@graphene, α-Fe@graphene and ε-Fe_2C@graphene. **a,** C 1*s*, and **b,** N 1*s* regions of θ-Fe_3C@graphene. **c,** C 1*s*, and **d,** N 1*s* regions of α-Fe@graphene. **e,** C 1*s*, and **f,** N 1*s* regions of ε-Fe_2C@graphene. I_1 denotes pyridine-like nitrogen (centered at 398.4 eV), I_2 denotes pyrrole-like nitrogen (centered at 399.7 eV), I_3 denotes "graphitic" nitrogen (centered at 401.0 eV), I_4 denotes "oxidized" nitrogen (centered at 402.6 eV).

Supplementary Table 3 Comparison of the activities of ε-Fe_2C@graphene with those of iron catalysts previously reported in the literature

Catalysts	CO conv. (%)	FTY ($\mu mol_{CO}\ g_{Fe}^{-1}\ s^{-1}$)	Reaction conditions	Ref.
ε-Fe_2C@graphene (Fe: 40.5 wt.%)	48.0	1,258	340 °C, 1.0 MPa, H_2/CO = 1, 160 L g_{cat}^{-1} h^{-1}	This work
Fe/NG (Fe: 8.0 wt.%)	1.4	17	340 °C, 0.5 MPa, H_2/CO = 1, 8 L g_{cat}^{-1} h^{-1}	6
Fe/CNT (Fe: 12.0 wt.%)	88.0	30	340 °C, 2.0 MPa, H_2/CO = 1, 1.5 L g_{cat}^{-1} h^{-1}	7
Fe-Cu-K-SiO_2 (Fe: 32.0 wt.%)	79.0	11	340 °C, 2.0 MPa, H_2/CO = 1, 1.5 L g_{cat}^{-1} h^{-1}	7
Fe/α-Al_2O_3 (Fe: 6.0 wt.%)	77.0	85	340 °C, 2.0 MPa, H_2/CO = 1, 1.5 L g_{cat}^{-1} h^{-1}	7
20Fe/N-CNT (Fe: 17.7 wt.%)	48.3	570	340 °C, 2.5 MPa, H_2/CO = 1, 50 L g_{cat}^{-1} h^{-1}	8
Fe@C (Fe: 25.0 wt.%)	59.0	490	340°C, 2.0 MPa, H_2/CO = 1, 60 L g_{cat}^{-1} h^{-1}	9
Fe_5C_2@C (Fe: 20.0 wt.%)	47.0	520	320 °C, 1.5 MPa, H_2/CO = 1, 60 L g_{cat}^{-1} h^{-1}	10
Fe/AC (Fe: 20.0 wt.%)	4.9	7	320 °C, 1.5 MPa, H_2/CO = 1, 8 L g_{cat}^{-1} h^{-1}	10

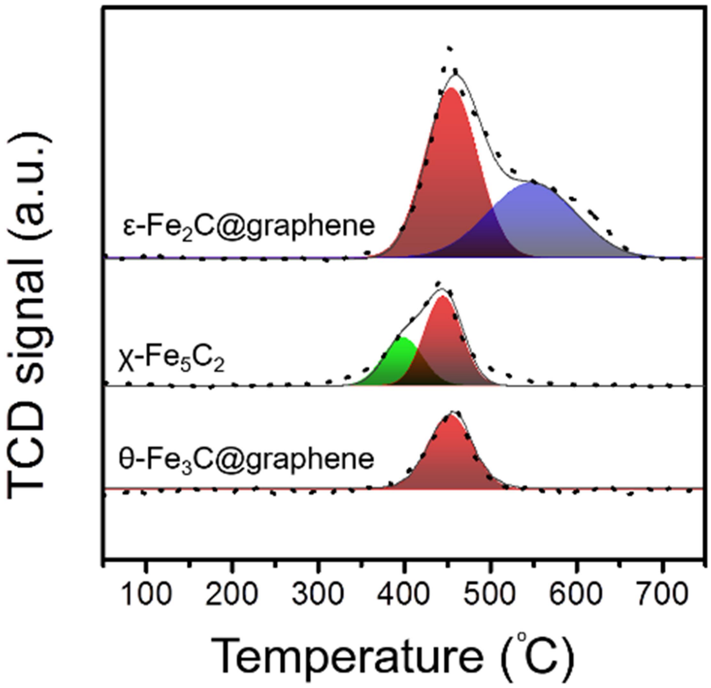

Supplementary Figure 7 CO-TPD profiles of the different iron catalysts.

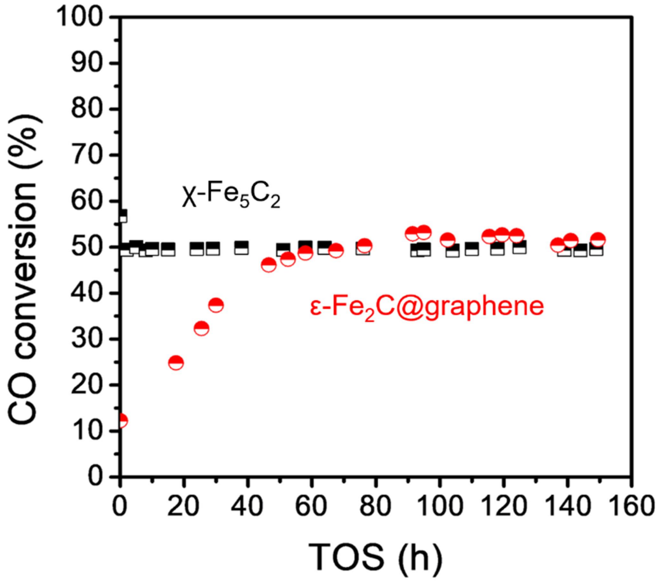

Supplementary Figure 8 Time-on-steam evolution of CO conversion for ε-Fe_2C and χ-Fe_5C_2 at the same conversion (~50 %) with different GHSVs. Reaction conditions: H_2/CO = 1/1 (mol/mol), 573 K, p = 10 bar, GHSV of 16.0 L g_{cat}^{-1} h^{-1} for χ-Fe_5C_2 while 64.0 L g_{cat}^{-1} h^{-1} for ε-Fe_2C@graphene.

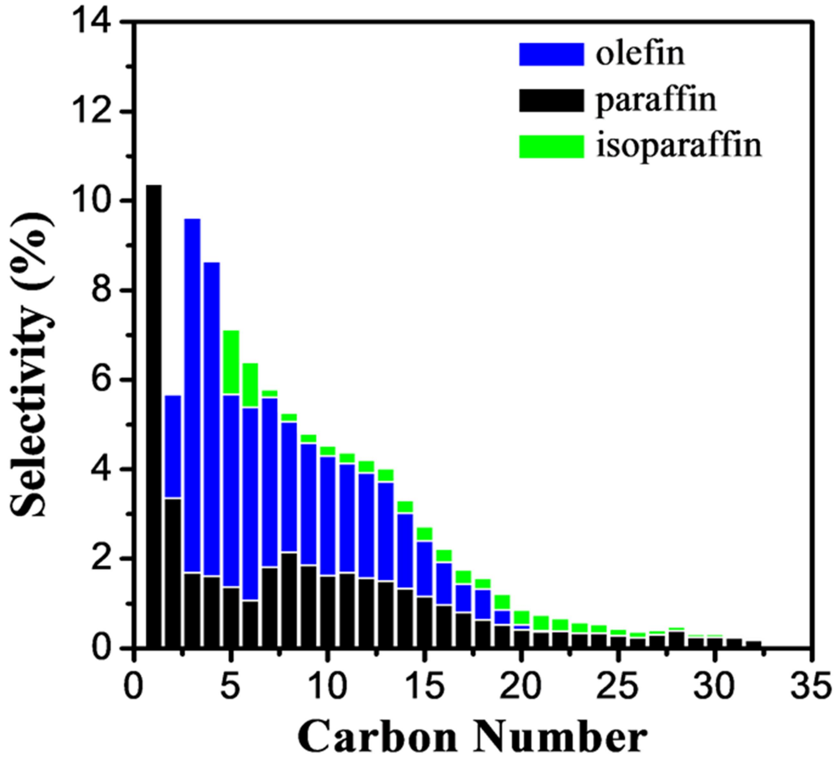

Supplementary Figure 9 Product distribution for Fischer-Tropsch synthesis over ε-Fe_2C@graphene catalyst. Reaction condition: H_2/CO = 1/1 (mol/mol), 573 K, GHSV = 64.0 L g_{cat}^{-1} h^{-1}, p = 10 bar.

Supplementary Table 4 Averaged surface energy (γ, eV/Å^2) referring to the carbon potential (μ_C = -7.62 ~ -7.53 eV) of the Fe_2C surfaces with different termination and thickness

Fe_2C surfaces	*k*-point	termination	thickness (Å)	γ (eV/Å^2)	deviation[a]
(1-21)-*p*(1×1)	5 × 5 × 1	stoichiometric	10.6	0.1081	0.01%
			13.0	0.1081	
		C-rich	11.8	0.1549	0.05%
			14.1	0.1549	
		Fe-rich	13.0	0.2255	0.01%
			15.3	0.2254	
(2-21)-*p*(1×1)	3 × 5 × 1	stoichiometric	9.7	0.1326	0.37%
			11.8	0.1321	
		C-rich	10.5	0.1149	0.29%
			12.6	0.1146	
		Fe-rich	10.9	0.1497	0.21%
			13.0	0.1500	
(101)-*p*(1×1)	5 × 5 × 1	stoichiometric	12.0	0.1297	0.12%
			15.2	0.1299	
		C-rich	10.1	0.0886	0.12%
			13.2	0.0887	
		Fe-rich	13.2	0.2018	0.04%
			15.8	0.2019	

[a] Relative deviation of surface energy is calculated by $\frac{|\gamma(\text{thicker}) - \gamma(\text{thinner})|}{\gamma(\text{thinner})}$%.

Supplementary Table 5 Lattice mismatch of the graphene(-N) layer to the Fe_2C supercells evaluated by the relative deviation of area (*A*)

Fe_2C surfaces	*k*-point	Fe_2C	graphene		graphene-N	
		A (Å^2)	*A* (Å^2)	mismatch[a]	*A* (Å^2)	mismatch[a]
(1-21)-*p*(1×1)	5 × 5 × 1	48.05	42.64	11.27%	42.45	11.66%
(2-21)-*p*(1×2)	3 × 3 × 1	107.58	105.66	1.91%	105.38	2.04%
(101)-*p*(1×2)	5 × 3 × 1	71.53	63.69	10.96%	63.44	11.30%

[a] Lattice mismatch of the graphene(-N) layer to the Fe_2C is calculated by $\frac{|A(\mathrm{Fe_2C}) - A(\mathrm{graphene})|}{A(\mathrm{Fe_2C})}\%$ or $\frac{|A(\mathrm{Fe_2C}) - A(\mathrm{graphene\text{-}N})|}{A(\mathrm{Fe_2C})}\%$.

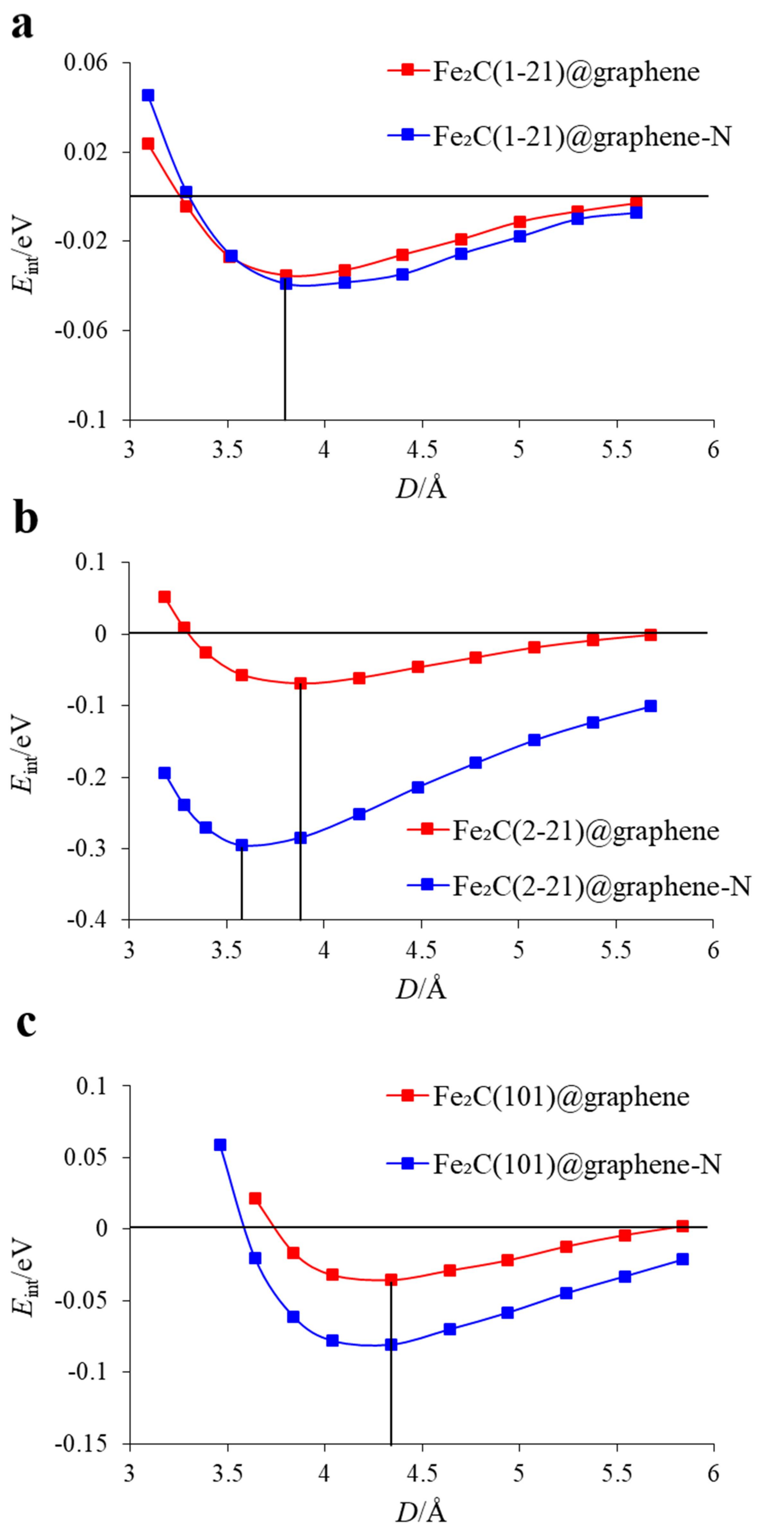

Supplementary Figure 10 Interaction energy evaluated by E_{int} = E(Fe_2C@graphene(-N)) – E(Fe_2C) – E(graphene(-N)) as a function of the distance (D) between Fe_2C and graphene(-N).

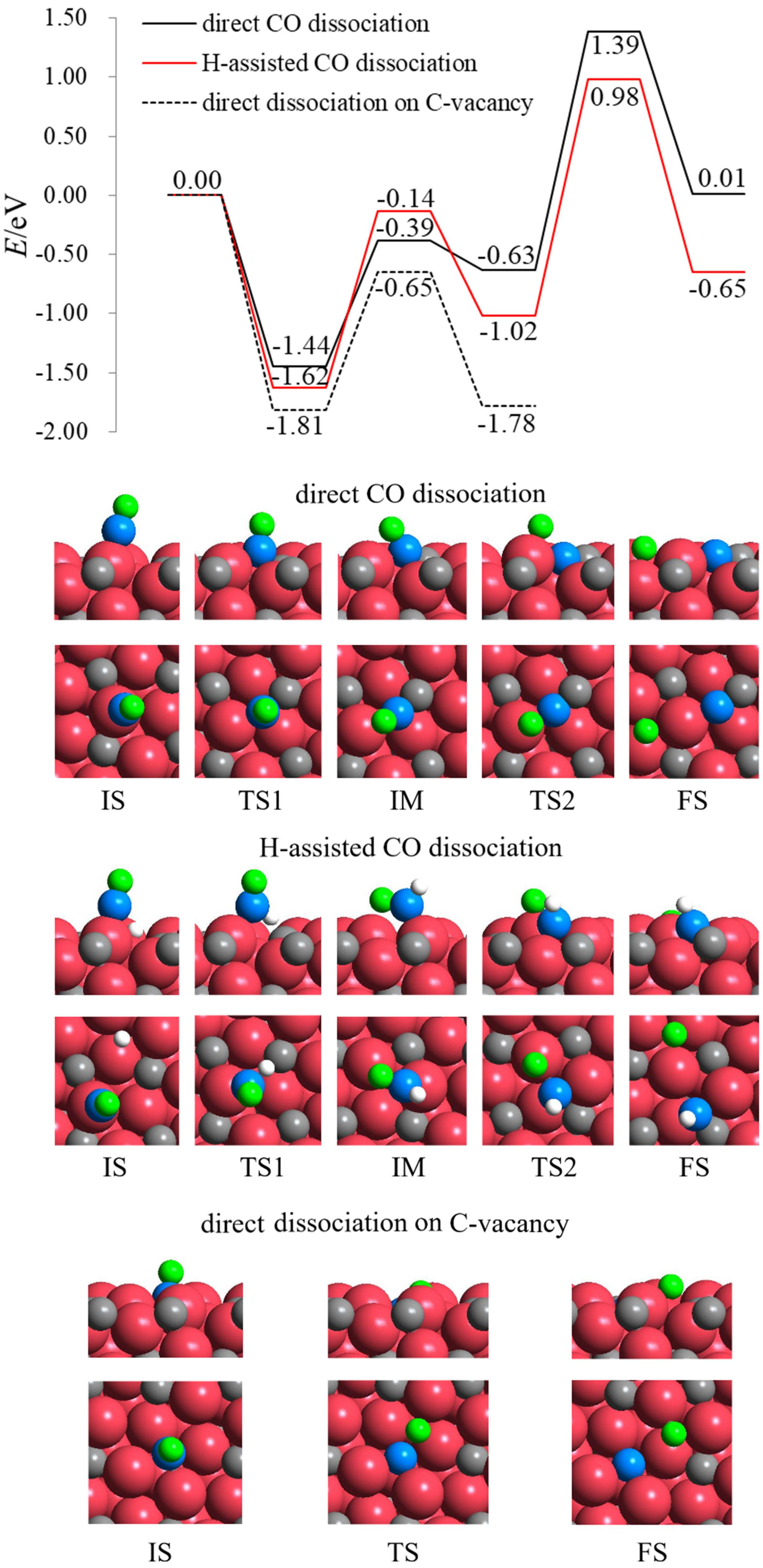

Supplementary Figure 11 Reaction energy profiles and structures of CO dissociation on Fe_2C(1-21) (Fe: red; C: grey; C of CO: blue; O: green; H: white).

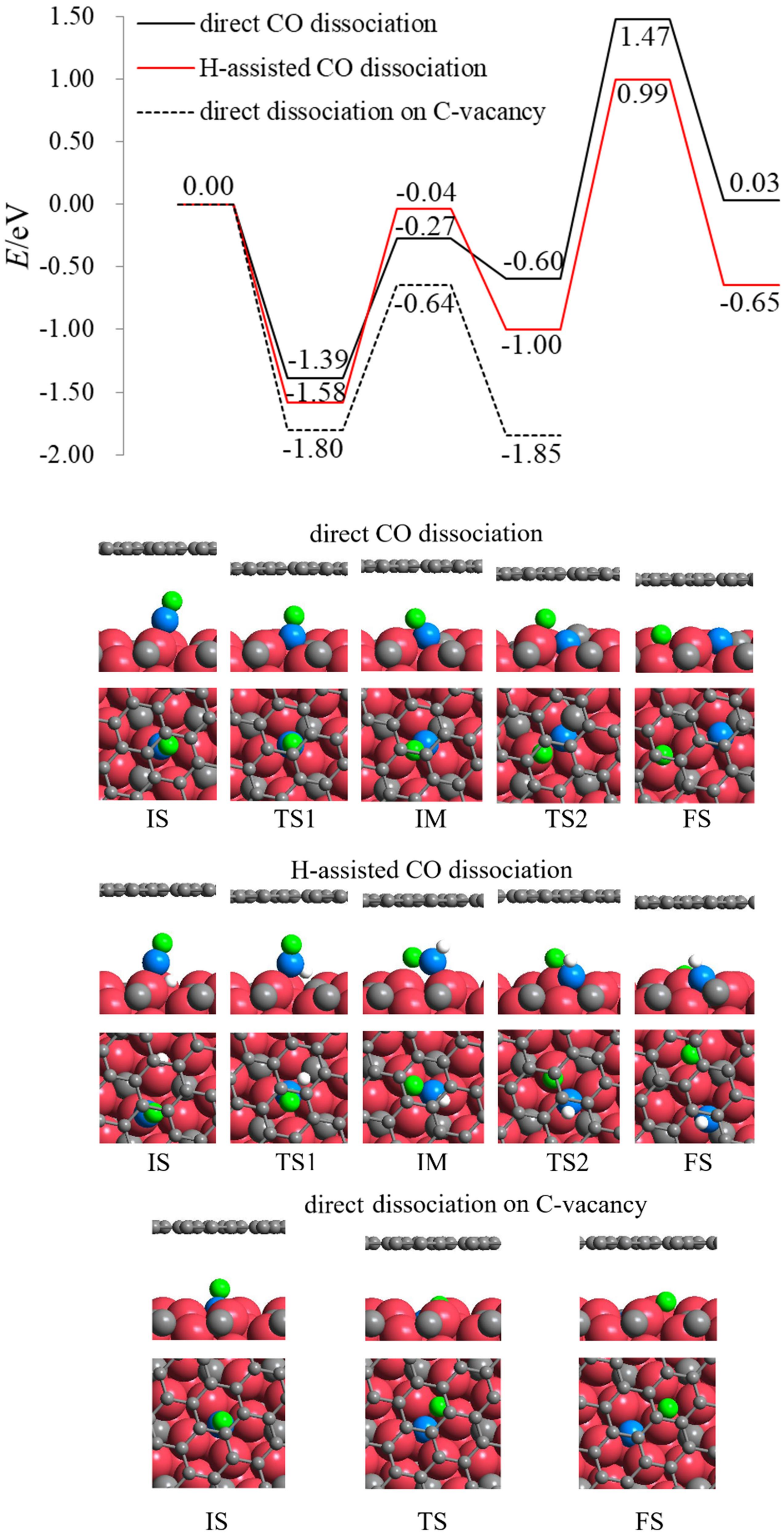

Supplementary Figure 12 Reaction energy profiles and structures of CO dissociation on Fe_2C(1-21)@graphene (Fe: red; C: grey; C of CO: blue; O: green; H: white).

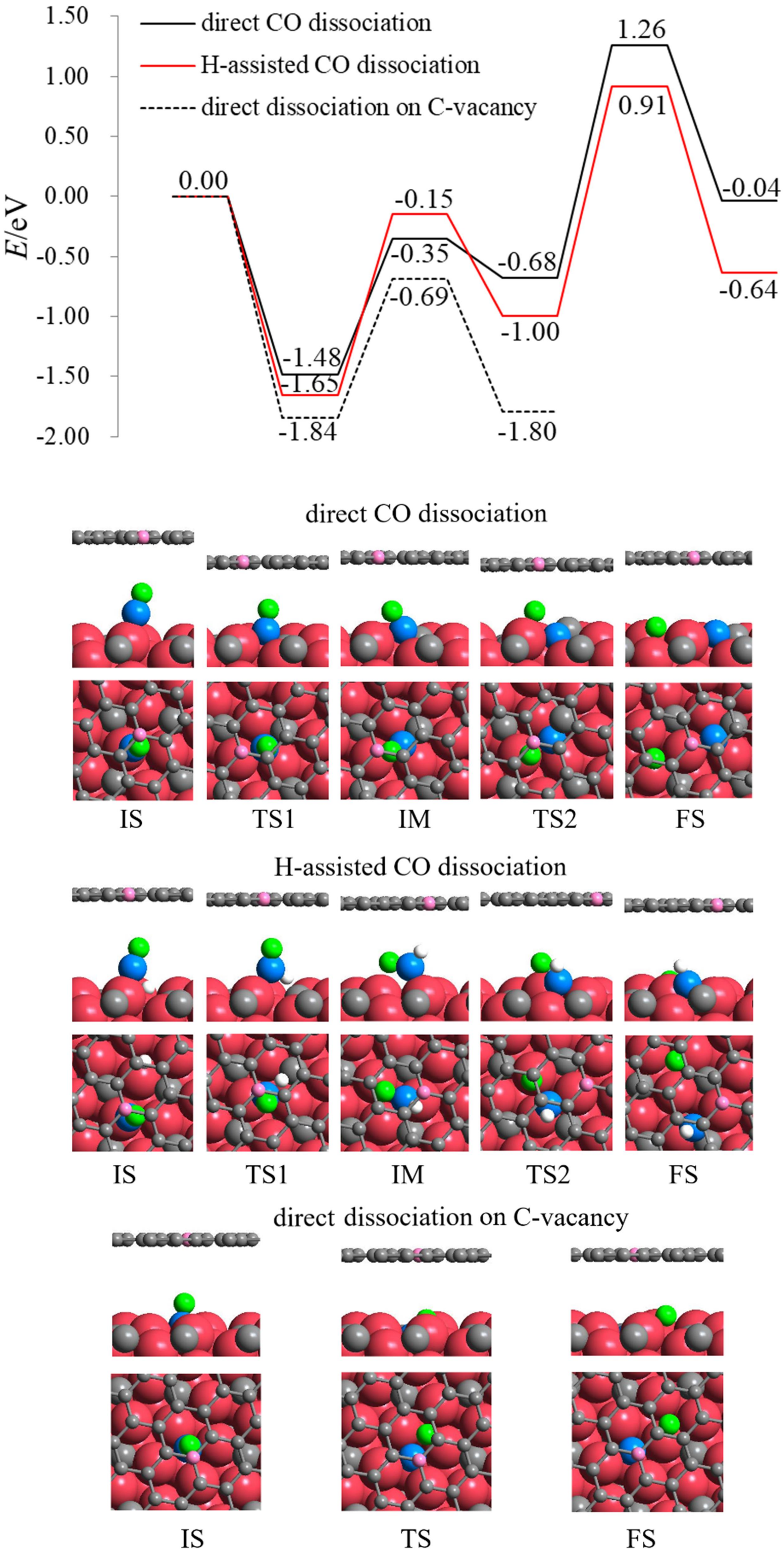

Supplementary Figure 13 Reaction energy profiles and structures of CO dissociation on Fe_2C(1-21)@graphene-N (Fe: red; C: grey; C of CO: blue; O: green; H: white; N: pink).

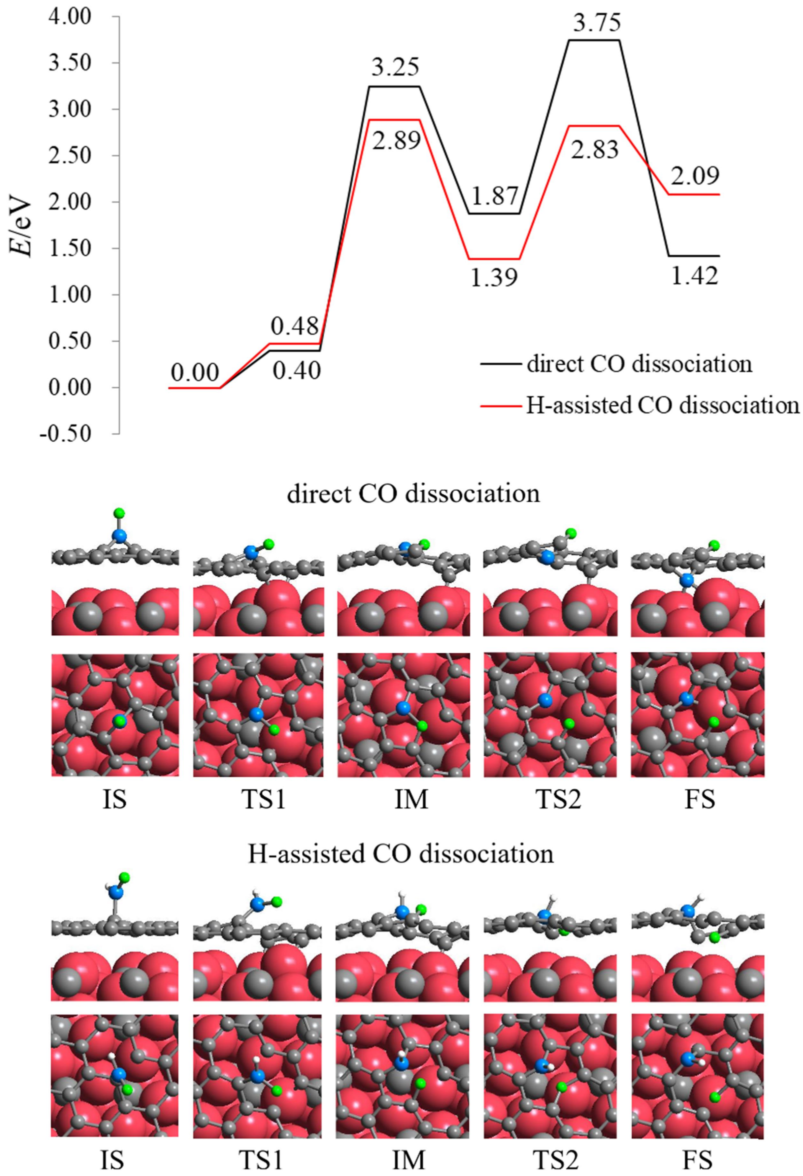

Supplementary Figure 14 Reaction energy profiles and structures of CO dissociation on graphene/Fe_2C(1-21) (Fe: red; C: grey; C of CO: blue; O: green; H: white).

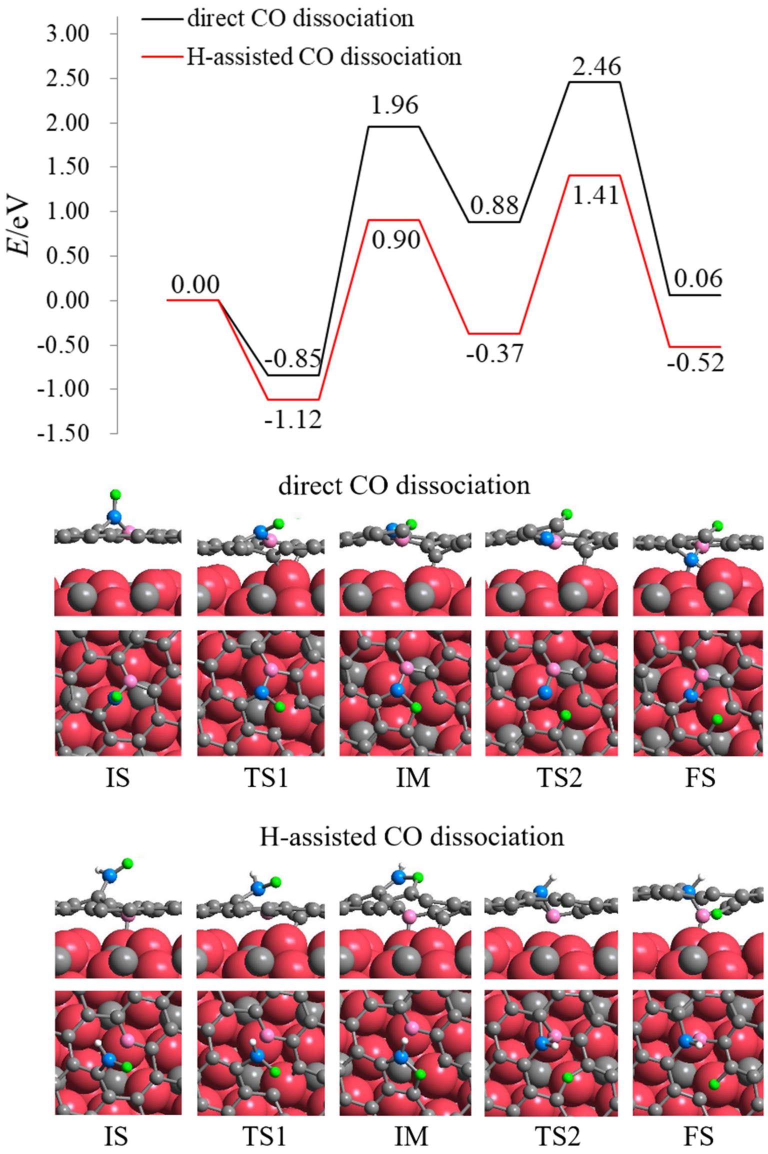

Supplementary Figure 15 Reaction energy profiles and structures of CO dissociation on graphene-N/Fe_2C(1-21) (Fe: red; C: grey; C of CO: blue; O: green; H: white; N: pink).

Supplementary References

1. Zhao, S. et al. Determining surface structure and stability of ε-Fe_2C, χ-Fe_5C_2, θ-Fe_3C and Fe_4C phases under carburization environment from combined DFT and atomistic thermodynamic studies. *Catal. Struct. React.* **1**, 44-59 (2015).
2. Kresse, G. & Furthmüller, J. Efficient iterative schemes for ab initio total-energy calculations using a plane-wave basis set. *Phys. Rev. B* **54**, 11169-11186 (1996).
3. Frisch, M. J. et al. *Gaussian 09, Revision D.01*, Gaussian, Inc., Wallingford CT, 2013.
4. http://www.grc.nasa.gov/WWW/CEAWeb/ceaWhat.htm.
5. de Smit, E. et al. Stability and reactivity of ε-χ-θ iron carbide catalyst phases in Fischer-Tropsch synthesis: controlling μ_c. *J. Am. Chem. Soc.* **132**, 14928-14941 (2010).
6. Chen, X., Deng, D., Pan, X., Hu, Y. & Bao, X. N-doped graphene as an electron donor of iron catalysts for CO hydrogenation to light olefins. *Chem. Commun.* **51**, 217-220 (2015).
7. Torres Galvis, H. M. et al. Supported iron nanoparticles as catalysts for sustainable production of lower olefins. *Science* **335**, 835-838 (2012).
8. Schulte, H. J., Graf, B., Xia, W. & Muhler, M. Nitrogen- and oxygen-functionalized multiwalled carbon nanotubes used as support in iron-catalyzed, high-temperature Fischer-Tropsch Synthesis. *ChemCatChem* **4**, 350-355 (2012).
9. Santos, V. P. et al. Metal organic framework-mediated synthesis of highly active and stable Fischer-Tropsch catalysts. *Nat. Commun.* **6**, 6451-6458 (2015).
10. Hong, S. Y. et al. A new synthesis of carbon encapsulated Fe_5C_2 nanoparticles for high-temperature Fischer-Tropsch synthesis. *Nanoscale* **7**, 16616-16620 (2015).
